# Association of Health Literacy and Area Deprivation With Initiation and Completion of Telehealth Visits in Adult Medicine Clinics Across a Large Health Care System

**DOI:** 10.1001/jamanetworkopen.2022.23571

**Published:** 2022-07-25

**Authors:** Sarah H. Brown, Michelle L. Griffith, Sunil Kripalani, Sara N. Horst

**Affiliations:** 1 Vanderbilt University School of Medicine, Nashville, Tennessee; 2 Division of Diabetes, Endocrinology, and Metabolism, Department of Medicine, Vanderbilt University Medical Center, Nashville, Tennessee; 3 Division of General Internal Medicine and Public Health, Department of Medicine, Vanderbilt University Medical Center, Nashville, Tennessee; 4 Division of Gastroenterology, Hepatology, and Nutrition, Department of Medicine, Vanderbilt University Medical Center, Nashville, Tennessee

## Abstract

This cross-sectional study assesses whether health literacy and area deprivation index are associated with completion rates of telehealth video visits among adult patients in a large health care system.

## Introduction

As telehealth continues to evolve, the interface type becomes more important. Audio-only visits are associated with lower patient satisfaction and health understanding, whereas telehealth insurance coverage increasingly requires video interface.^1^ Previous investigations^1,2,3^ have found that age, insurance status, and race and ethnicity may affect video telehealth visit completion rates. However, understanding of how social determinants of health affect health technology use is limited. This study assessed whether health literacy and area deprivation index (ADI) are associated with completion rates of telehealth video visits.^1,4^

## Methods

This single-center cross-sectional study of scheduled telehealth visits included all adult medicine outpatient clinics across a large health care system during the COVID-19 pandemic (March 20 to July 30, 2020); visits during this period were telehealth only, eliminating preference bias. Billing codes captured a scheduled telehealth visit with an absentee patient (no-show), a video-conferencing, or an audio-only visit. Visits were first attempted with video but could be converted to audio only if video failed. Patients who completed health literacy questionnaires using the validated 3-item Brief Health Literacy Screen (BHLS) were included.^5^ Self-reported patient address mapped to neighborhood socioeconomic data generated the ADI.^6^ Race and ethnicity data were obtained from self-reported electronic medical record data and are relevant because prior studies^1,3^ have shown that race and ethnicity are possible risk factors for worse telehealth outcomes. This study was approved by the Vanderbilt University Medical Center Institutional Review Board, which waived informed consent for retrospective use of data. This study followed the STROBE reporting guideline.

Statistical analysis included Wilcoxon rank sum (continuous variables) and Pearson χ^2^ (categorical variables) testing. Multivariable logistic regression models evaluated the likelihood of a no-show and video vs audio-only visit, adjusting for patient age, sex, race and ethnicity, insurance status, low health literacy (BHLS score ≤9),^5^ and ADI. Statistical analysis was completed using Stata, version 16.1 (StataCorp LLC). Two-sided *P* < .05 indicated statistical significance.

## Results

Of 18 130 scheduled telehealth visits, 675 (4%) were no-shows. Patient characteristics included a median age of 60 (range, 18-101) years, with 10 454 women (58%) and 7676 men (42%). In terms of race and ethnicity, 440 patients (2%) were Asian, 2672 (15%) were Black, 181 (1%) were Hispanic, and 14 882 (82%) were White. Among all patients, 8733 (48%) had noncommercial health insurance. Of 17 455 completed visits, 1375 (8%) were audio only. Patients with audio-only visits exhibited a significantly lower BHLS score (mean [SD], 13.1 [2.6] vs 13.7 [2.1]) and higher ADI (mean [SD], 0.33 [0.12] vs 0.30 [0.12]) than patients who completed a video visit (*P* < .001 for both) (Table 1).

**Table 1.** Baseline Characteristics of Completed Telehealth Visit Participants by Video Visit Compared to Failed Video (Audio Only) Visit

Characteristic	Type of visit		*P* value^a^
	Audio-only (failed video) (n = 1375)	Video (n = 16 080)	
Patient age, median (range), y	63 (48-78)	53 (36-70)	<.001
Sex, No. (%)			
Women	1003 (73)	9648 (60)	<.001
Men	372 (27)	6432 (40)	<.001
Noncommercial insurance, No. (%)	866 (63)	5628 (35)	<.001
Race and ethnicity, No. (%)			
Asian	14 (1)	319 (2)	.02
Black	308 (22)	2261 (14)	<.001
Hispanic	13 (1)	205 (1)	.12
White	1040 (75)	13 295 (83)	<.001
BHLS score, mean (SD)^b^	13.1 (2.6)	13.7 (2.1)	<.001
Area deprivation index, mean (SD)	0.33 (0.12)	0.30 (0.12)	<.001

Abbreviation: BHLS, Brief Health Literacy Screen.

^a^ Wilcoxon rank sum testing was used for continuous variables and the Pearson χ^2^ test was used for categorical variables.

^b^ Scores of 9 or less indicated low health literacy.

In multivariable analysis, low health literacy was not associated with overall telehealth no-shows (odds ratio [OR], 0.90 [95% CI, 0.61-1.34]). Higher ADI was associated with higher no-show likelihood (OR, 2.28 [95% CI, 1.12-4.65]) (Table 2).

**Table 2.** Risk Factors for No-Show and Failed Video Telehealth Visits

Characteristic	Type of visit^a^			
	No-show		Audio-only (failed video)	
	OR (95% CI)	*P* value	OR (95% CI)	*P* value
Age, y				
18-30	1 [Reference]	NA	1 [Reference]	NA
31-50	0.80 (0.56-1.14)	.22	1.56 (1.16-2.10)	.003
51-70	0.81 (0.57-1.14)	.23	3.21 (2.41-4.28)	<.001
>70	0.72 (0.48-1.07)	.11	4.63 (3.42-6.27)	<.001
Sex				
Men	1 [Reference]	NA	1 [Reference]	NA
Women	1.20 (1.02-1.41)	.03	1.04 (0.95-1.13)	.41
Insurance status				
Commercial	1 [Reference]	NA	1 [Reference]	NA
Medicare	1.23 (1.004-1.51)	.046	1.84 (1.65-2.04)	<.001
Medicaid	1.97 (1.34-2.89)	.001	2.20 (1.71-2.83)	<.001
Self-pay	0.71 (0.31-1.61)	.41	1.67 (1.18-2.37)	.004
Race and ethnicity				
Asian	0.63 (0.30-1.34)	.23	0.68 (0.47-1.00)	.05
Black	0.98 (0.78-1.24)	.86	1.69 (1.51-1.89)	<.001
Hispanic	1.69 (0.95-2.98)	.07	0.97 (0.64-1.49)	.90
White	1 [Reference]	NA	1 [Reference]	NA
Area deprivation index	2.28 (1.12-4.65)	.02	6.78 (4.69-9.79)	<.001
Low health literacy^b^	0.90 (0.61-1.34)	.62	1.40 (1.17-1.67)	<.001

Abbreviations: NA, not applicable; OR, odds ratio.

^a^ Multivariable logistic regression models were adjusted for patient age, sex, race and ethnicity, insurance status, low health literacy, and area deprivation index.

^b^ Defined by Basic Health Literacy Screen score of 9 or less.

Age, insurance status, and identifying as Black increased the likelihood of an audio-only visit, consistent with prior research.^3^ Low health literacy (OR, 1.40 [95% CI, 1.17-1.67]) and higher ADI (OR, 6.78 [95% CI, 4.69-9.79]) were also independently associated with audio-only visits (Table 2).

## Discussion

In this study, factors from prior work (age, insurance status, race and ethnicity) remained significant,^3^ but low health literacy and higher ADI were also associated with video conversion to audio-only interface—an important distinction owing to increasing insurance coverage requirements for video telehealth visits. Area deprivation index showed the strongest likelihood of audio-only connection. The BHLS score also showed a higher likelihood of audio-only interface; although not a specific measure of digital literacy, it may reflect patients’ ability to follow video conferencing instructions.^5^ Study limitations include performance at a single institution and patient sample with relatively high levels of health literacy, affecting generalizability.

This study further defines risk factors that are associated with telehealth access. Although barriers such as wireless internet access, technology cost, and privacy will require societal changes, health care systems should consider ways to improve telehealth access. Previous work has shown that interventions (eg, a previsit telephone call) can improve video telehealth completion.^3^ Future directions include electronic medical record triggers that identify patients at risk for telehealth failure, study of ADI components, and association of health literacy and patient portal use.
